# Toxicological Studies of ^212^Pb Intravenously or Intraperitoneally Injected into Mice for a Phase 1 Trial

**DOI:** 10.3390/ph8030416

**Published:** 2015-07-24

**Authors:** Diane E. Milenic, Alfredo A. Molinolo, María S. Solivella, Eileen Banaga, Julien Torgue, Sarah Besnainou, Martin W. Brechbiel, Kwamena E. Baidoo

**Affiliations:** 1 Radioimmune & Inorganic Chemistry Section, Radiation Oncology Branch, Center for Cancer Research, National Cancer Institute, National Institutes of Health, Bethesda, MD 20892, USA; E-Mails: sarah.besnainou@gmail.com (S.B.); martinwb@mail.nih.gov (M.W.B.); baidook@mail.nih.gov (K.E.B.); 2 Oral and Pharyngeal Cancer Branch, National Institute of Dental and Craniofacial Research, National Institutes of Health, Bethesda, MD 20892, USA; E-Mails: amolinol@mail.nih.gov (A.A.M.); solivellams@yahoo.com (M.S.S.); 3 Areva Med LLC, 4800 Hampden Lane, Suite 200, Bethesda, MD 20817, USA; E-Mails: Eileen.banaga@areva.com (E.B.); Julien.torgue@areva.com (J.T.)

**Keywords:** Pb-212, FDA toxicity study, radioimmunotherapy, targeted alpha-radiation

## Abstract

Faced with the novelty of a ^212^Pb-labeled monoclonal antibody (mAb) for clinical translation, concerns were expressed by the Food and Drug Administration (FDA) regarding ^212^Pb prematurely released from the mAb-chelate conjugate. The objective of this study was to simulate the worst case scenario of such a failure. Groups of Balb/c mice (*n* = 9–20) were administered ^212^Pb by intraperitoneal (0.0925–1.85 MBq) or intravenous (0.0925–1.11 MBq) injection and then euthanized at 7 or 90 days to assess acute or chronic effects. Weights were recorded prior to injection of the ^212^Pb and at the end of the observation periods. Blood samples were collected for clinical chemistry and blood cell analysis. Thirty tissues were harvested and formalin fixed for histopathological examination. Treatment related effects of the ^212^Pb were observed in the bone marrow, spleen, kidneys and the liver. Histological alterations in these organs were considered mild to moderate, indicating low grade toxicity, and not considered severe enough to affect function. This data was presented to the FDA and determined to be acceptable. The clinical trial with ^212^Pb-TCMC-trastuzumab was approved in January 2011 and the trial opened at the University of Alabama at Birmingham (UAB) in July.

## 1. Introduction

The radionuclide, ^212^Pb, has been a focus of investigation for radioimmuno-therapy (RIT) [1,2,3,4,5,6,7,8,9,10,11,12,13] as well as a supply of cytotoxic α-particles from the decay of its ^212^Bi daughter, and the strategy of using the ^212^Pb as an *in vivo* generator for ^212^Bi circumvents the logistical difficulties of working directly with the short-lived ^212^Bi (T_½_ 60.6 min). The 10.6 h half-life of ^212^Pb also extends the time to deliver and target tumors with ^212^Bi. This results in a greater therapeutic impact and reduces the dose required for an effective therapeutic benefit. At the same time toxicity to normal tissues is reduced. An important pre-requisite to the success of ^212^Pb as a candidate for RIT was the development of an improved bifunctional chelate for sequestering Pb(II) [14].

The preclinical efficacy of ^212^Pb-labeled mAb (trastuzumab) has been clearly demonstrated and the results of these studies have now been translated to the clinic [3,4,5,6]. In 2011, a phase I clinical trial (NCT01384253), sponsored by AREVA Med LLC (Bethesda, MD, USA), was initiated at the UAB to determine the toxicity profile of ²¹²Pb-TCMC-trastuzumab, its dose-limiting toxicities, and its anti-tumor effects in patients. Patients eligible for the trial were those with HER-2 expressing intraperitoneal carcinomatosis (e.g., ovarian, pancreatic, colon, gastric, endometrial, or breast) who had failed standard therapies. Tumors were required to have either a score of at least 1+ by immunohistochemistry in more than 10% of the cells or have demonstrated HER-2 amplification by fluorescent *in situ* hybridization, or the patient’s HER-2 serum levels had to be greater than 15ng/mL by ELISA. This was the first such human study of ^212^Pb-radioimmunotherapy.

The clinical trial is a culmination of studies beginning with the synthesis and characterization of 1,4,7,10-tetra-(2-carbamoyl methyl)-cyclododecane (TCMC) [14]. In acid dissociation experiments, TCMC was found to overcome the pH lability that was associated with DOTA. Subsequent to these studies was the *in vivo* demonstration of the therapeutic efficacy of ^212^Pb-trastuzumab for the treatment of disseminated peritoneal disease as a single modality as well as in combination with chemotherapy [2,3,4,5,6].

Prior to approval of a drug for evaluation in a clinical study, the FDA usually mandates some form(s) of a toxicology study of the drug. This is particularly salient when a novel agent, which in this case was the radionuclide, is a component of the drug. Logical expectations were that the final injectate into humans, ^212^Pb-TCMC-trastuzumab, on which there is significant literature, would be the agent for acute and chronic toxicity studies. However, with ^212^Pb being a truly unknown agent, a safety profile of the free radionuclide was one of the studies requested by the FDA. The rationale for this request was to ascertain the effects of ^212^Pb in a worst-case scenario in case a complete failure of the radiolabeled product occurred and the ^212^Pb dissociated from the chelate and subsequently localized in tissue. Defining the impact of toxicity to those tissues, identification of tissues at risk and activity limiting organs were critical considerations. Due to the unique nature of such settings and the execution *in vivo* of such studies with ^212^Pb, it was felt that the methods and results reported herein would be of interest and significance to investigators researching novel therapeutic radionuclides for medical applications.

The purpose of this report is to present that study. Various levels of ^212^Pb activity were administered via intraperitoneal (i.p.) or intravenous (i.v.) injection in BALB/c mice. The mice were euthanized at 7 or 90 days to assess the acute and chronic effects, respectively. The i.v. injection route was requested by the FDA as part of the study despite the fact that i.v. administration of the ^212^Pb-TCMC-trastuzumab was not planned.

## 2. Results

### 2.1. Mortality of Normal Balb/c Mice Injected i.p. or i.v. with ^212^Pb

No deaths occurred in any of the groups receiving 0.0925, 0.185, 0.278 or 0.370 MBq by either i.p. or i.v. injection route (Table 1). Two deaths were noted at 16 days in the 0.740 MBq i.v. injected 90 days group. In those mice that received the ^212^Pb by i.p. injection, deaths occurred in the 0.555 MBq (at 11, 40 and 90 days), 1.11 MBq (at 69 days), 1.488 MBq (at 8, 11 days and 1 unrecorded) and 1.85 MBq (3 mice at 9 days) groups. To expedite the study and due to the logistics of conducting the study, mice in the 90 days groups were injected with the ^212^Pb first, the 7 days groups were injected at later times. As a result of deaths, along with hematology and blood chemistry data, observed in the 90 days groups receiving 1.488 and 1.850 MBq by i.p. injection, and when the hematology and blood chemistry data was considered, the decision was made to not further subject the mice to the higher levels of radioactivity, so no data was collected for these two groups.

**Table 1:** Mice surviving to scheduled time of euthanasia.

Activity (MBq)	Necropsy Groups				Hematology Groups	
	i.v.		i.p.		i.v.	i.p.
	Day 7	Day 90	Day 7	Day 90	Day 90	
0.0925	5/5	5/5	5/5	5/5	5/5	5/5
0.185	10/10	5/5	5/5	5/5	5/5	5/5
0.278	5/5	5/5	5/5	5/5	5/5	5/5
0.370	5/5	10/10	5/5	10/10	5/5	5/5
0.555	5/5	5/5	5/5	7/10	5/5	5/5
0.740	5/5	3/5	5/5	5/5	5/5	5/5
1.110	5/5	5/5	5/5	4/5	5/5	4/5
1.488			ND ^a^	1/4		3/5
1.850			ND	2/5		0/5

^a^ The i.p. injections of 1.488 and 1.850 MBq of ^212^Pb were not performed for the 7 days timepoint. (ND, No data).

Among the mice utilized for the hematology studies, all of the mice injected with ^212^Pb by the i.v. route survived the 90 days study period. In contrast, among those that were injected i.p., deaths occurred at 16 days (1 mouse) in the 1.110 MBq group; 1 mouse each at 10 and 16 days in the 1.488 MBq group. Among the mice injected at the 1.850 MBq level, deaths were noted at 7 (3 mice), 10 (1 mouse) and 16 days (1 mouse).

### 2.2. Effect of ^212^Pb Given i.p. or i.v. on the Body Weight of Normal Balb/c Mice

Pre-study body weights of the mice were recorded before injection with the ^212^Pb solution, then at 7 and 90 days just prior to euthanasia (Table 2). The weights of untreated mice recorded at 7 and 90 days were provided for comparison. Among the mice that received ^212^Pb by i.p. administration, the greatest weight loss observed at 7 days occurred in the 1.110 MBq group (9.8%). This weight loss was found to be significant when compared to the control mice (*p* < 0.001) or to the 0.0925 MBq group (*p* = 0.015). Weight loss was also noted in the groups injected with 0.370, 0.555 and 0.740 MBq. Meanwhile, at 90 days, a net weight gain in all of the groups, ranging from 0.6 to 7.4 g (2.8 to 33.6%), was observed. For comparative purposes, untreated mice demonstrated an average weight gain of 20.4%. Among the groups of mice given the ^212^Pb by i.v. injection, weight loss was observed at 0.185, 0.370, 0.555, 0.740 and 1.110 MBq. At 90 days, the weights had not recovered in the two groups receiving the highest activity. The weights of the 0.740 and 1.110 MBq groups remained lower than the initial weights recorded at the beginning of the study. Meanwhile, the rest of the groups demonstrated a weight gain at the 90 day time point. Differences in the 7 day weights were significant only for the highest activity level of 1.110 MBq compared to 0.0925 MBq (*p* < 0.01), or to 0.278 MBq (*p* < 0.05). The difference for this high-activity group remained significant at day 90 (untreated *vs.* 1.110 MBq; *p* < 0.01). At both time points, the increasing level of activity (i.v.) paralleled the attenuation of weight gain.

**Table 2:** Body weights of Balb/c mice injected with ^212^Pb.

		Body Weight (g)							
Group		7 Days	90 Days	% Change					
Untreated		21.1 ± 2.1	25.4 ± 3.6	20.4					
MBq	Time Weighed	Body Weight (g)							
		I.P. Injected				I.V. Injected			
		7 Days	% Change	90 Days	% Change	7 Days	% Change	90 Days	% Change
0.0925	Initial ^a^	21.8 ± 1.1 ^b^	0.5	20.6 ± 0.8	13.1	26.1 ± 2.4	3.1	20.8 ± 1.5	12.0
	Final	21.9 ± 1.6		23.3 ± 0.9		26.9 ± 1.5		23.3 ± 0.9	
0.185	Initial	20.8 ± 1.6	2.4	21.2 ± 1.1	14.2	28.1 ± 3.0	−0.4	19.9 ± 1.1	17.6
	Final	21.3 ± 1.6		24.3 ± 1.5		28.0 ± 3.3		23.4 ± 2.1	
0.278	Initial	22.8 ± 1.6	0.9	21.3 ± 1.2	2.8	27.1 ± 2.8	0	20.8 ± 0.8	7.2
	Final	23.0 ± 1.5		21.9 ± 1.3		27.1 ± 2.2		22.3 ± 1.0	
0.370	Initial	26.7 ± 2.5	−9.7	21.8 ± 1.7	8.7	25.8 ± 1.0	−2.3	22.1 ± 0.9	3.6
	Final	24.1 ± 1.7		23.7 ± 2.1		25.2 ± 0.6		22.9 ± 0.8	
0.555	Initial	26.4 ± 2.9	−3.4	22.0 ± 1.2	4.5	25.6 ± 1.8	−3.1	20.2 ± 0.5	1.0
	Final	25.5 ± 3.3		23.0 ± 1.4		24.8 ± 2.1		20.4 ± 1.5	
0.740	Initial	21.9 ± 1.0	−6.4	24.8 ± 2.1	33.6	26.0 ± 1.8	−9.6	25.6 ± 2.0	−6.6
	Final	20.5 ± 0.8		32.2 ± 1.9		23.5 ± 0.5		23.9 ± 1.5	
1.110	Initial	25.4 ± 1.8	−9.8 *	19.7 ± 2.4	10.7	28.3 ± 2.0	−17.6 *	26.5 ± 1.9	−6.4 ^*^
	Final	22.9 ± 2.5		21.8 ± 2.5		23.3 ± 3.2		24.8 ± 1.6	
1.488	Initial	ND ^c^		23.5 ± 1.5	4.3				
	Final			24.5					
1.850	Initial	ND		24.2 ± 1.9					
	Final								

^a^ The mouse weights were recorded prior (initial) to intraperitoneal or intravenous injection of ^212^Pb and then at the time of euthanasia (final). ^b^ Values are the average body weight (g) and the standard deviation. ^c^ No data (ND) was available for these groups. * *p*-values are ≤ 0.05.

### 2.3. Hematological Analysis of Normal Balb/c Mice Injected i.p. or i.v. with ^212^Pb

**Figure 1:**
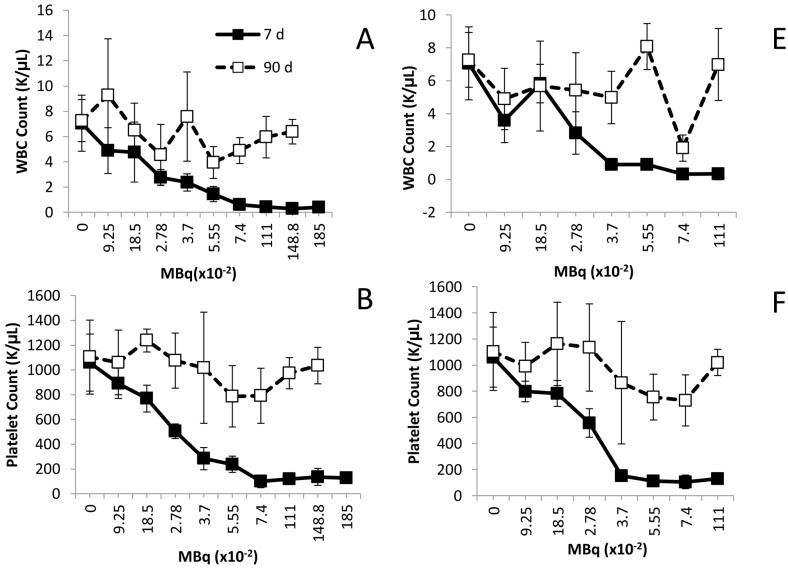

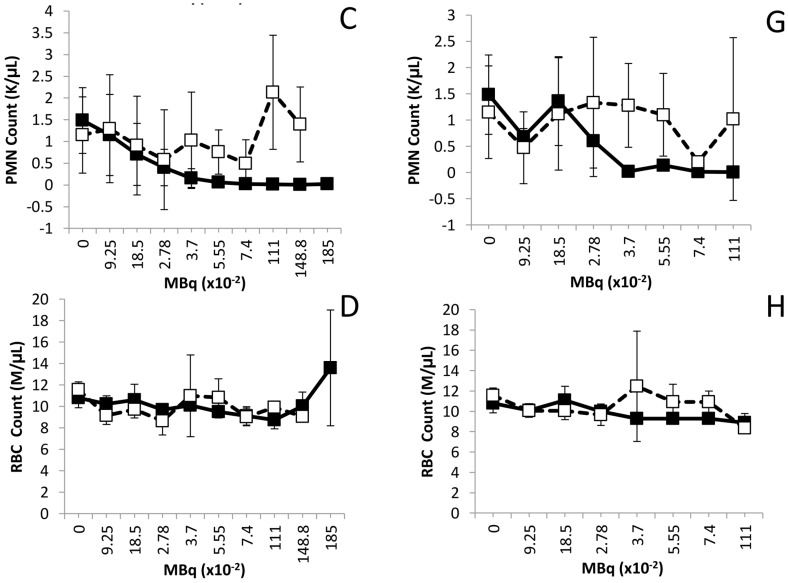
Effect of increasing levels of ^212^Pb activity on blood cell counts. Balb/c mice (*n* = 5) received increasing levels of ^212^Pb (MBq) by i.p. (Panels **A**–**D**) or i.v. (Panels **E**–**H**) injection. The effect on cell numbers are shown for WBC, platelets, PMN and RBC. The error bars represent the standard deviations.

Illustrated in Figure 1, activity-related decreases were noted in the mean values for white blood cells (WBC), platelets and polymorphonuclear leukocytes (PMN) at the 7 day time point following either i.v. or i.p. administration of the ^212^Pb. Differences in the WBCs of the groups injected i.p. with ^212^Pb (Figure 1A) were observed at 0.278 MBq and higher (*p* < 0.001). Compared to the untreated mice, the decreases in the WBC (Figure 1E) were significant in the i.v. injected mice at 0.0925 MBq (*p* < 0.01) and activity levels ≥ 0.278 MBq (*p* < 0.001). 

**Figure 2:**
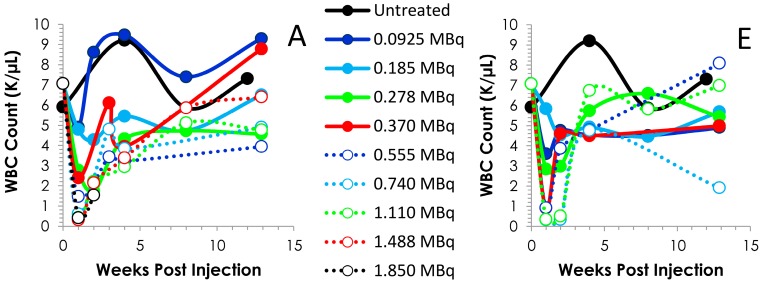

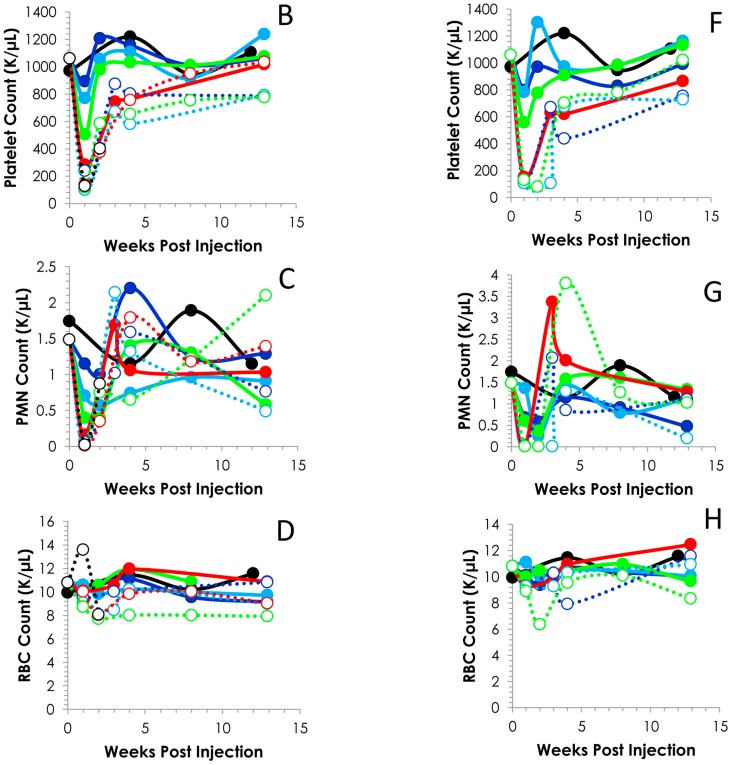
Time course of blood cell counts. Following the i.p. (Panels **A**–**D**) or i.v. (Panels **E**–**H**) injection of Balb/c mice (*n* = 5) with 0.0925 to 1.850 MBq of ^212^Pb, bloods were drawn weekly and analyzed. The values and the standard deviations are provided in Tables S1 and S2.

Significant differences were also identified in the platelet counts of the mice, comparing the treatment groups to the untreated group of mice. For the i.p injected mice, the p-values for the 0.185 MBq set was <0.05, and <0.001 for the 0.278 through to the 1.850 MBq groups (Figure 1B). In regards to the mice given i.v. injections (Figure 2F), differences were significant starting with the 0.0925 MBq group (*p* < 0.05). The 0.185 MBq group result also presented with a p value of 0.05 while the 0.278 to 1.110 MBq groups were *p* < 0.001.

PMN cells also exhibited an activity-dependent decrease in cell count in both the i.p. (Figure 2C) and the i.v. (Figure 1G) injected mice. In those mice that received the ^212^Pb solution by i.p. administration, only the 1.110 and 1.488 MBq groups returned to initial cell count levels by the 90 day time point. For the sets of mice injected i.v., with the exception of the 0.0925 and 0.740 MBq groups, the PMN cell count rebounded by 90 days, achieving levels equivalent to mice that did not receive ^212^Pb. There was a high degree of variance in the PMN values (large standard deviations) within groups and differences in PMN counts, compared to those of untreated mice, proved not significant (*p* > 0.05) in any of the groups.

None of the ^212^Pb activity levels, whether injected i.p. or i.v., had an effect on the red blood cell (RBC) count of the mice by the 7 day time point. Furthermore, no delayed effect on the red blood cell count was observed at the later time point of 90 days (Figure 1D,H).

Further analysis of the blood counts discussed above revealed typical patterns of recovery for the WBCs, platelets and PMNs (Figure 2). At all levels of radioactivity, i.p. and i.v. injected, within the experimental design of these studies, the nadir occurs *ca.* 7 days. Differences can be discerned between the i.p. and i.v. injected groups concerning the depletion and recovery of the cell counts. Also visualized in Figure 2 is the effect of the activity levels on the cell counts. The platelets of the 0.925 (*p* = 0.897), 0.185 (*p* = 0.151) and 0.278 (*p* = 0.109) MBq groups return to and/or exceed the count level before of the ^212^Pb while the 0.370 (14 days; *p* < 0.001) and 1.488 MBq (14 days, *p* = 0.002) groups do not return to pretreatment levels until 90 days, at which time the differences between the pretreatment and 90 day cell counts are insignificant which suggests recovery of the cell counts (*p* ≥ 0.532); the 0.555, 0.740 and 1.110 MBq groups attain a platelet count that is ~74% of the untreated group. Blood was collected for only the first two time points of the study from the group of mice that received 1.850 MBq of ^212^Pb by i.p. injection.

The pattern of recovery for the platelets from the mice injected i.v. was similar until the 0.740 and 1.110 MBq groups (Figure 2F). In these two groups, the nadir lasted through to 14 and 21 days, respectively, with recovery of the cell count occurring at the 28 d time point. Again, at the higher levels of ^212^Pb activity (0.370–1.110 MBq), the cell count does not attain the pre-injection level within 90 days. However, when compared to the untreated mice, the differences were not significant (0.370 MBq, *p* = 0641; 0.555 MBq, *p* = 0.364; 0.740 MBq, *p* = 0.059 and 1.110 *p* = 0.508). Values for other differential blood cell assays (absolute cell counts or percentages) were also variable (data not shown); it was not clear if these patterns reflected test article effects, mouse-to-mouse response variability, or other artifacts.

### 2.4. Effects of Increasing ^212^Pb Activity on Blood Chemistry

**Figure 3:**
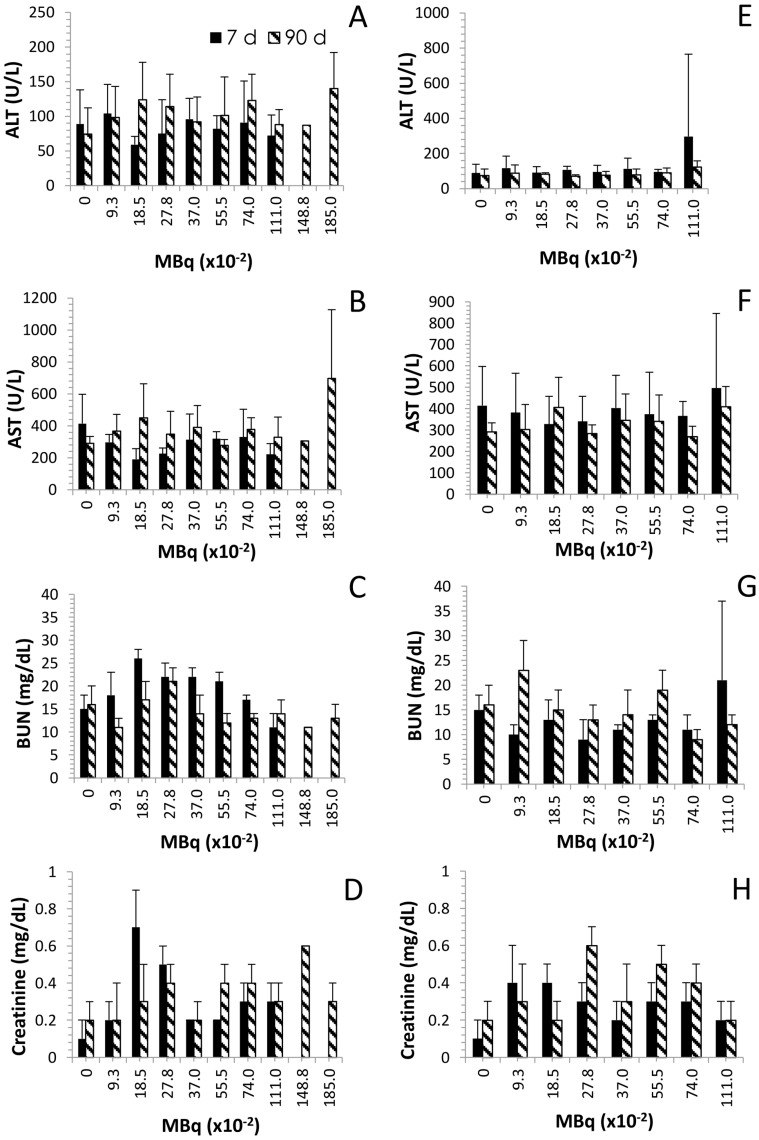
Effect of increasing levels of ^212^Pb activity on blood chemistry. Balb/c mice (*n* = 4–5) received increasing activity (MBq) of ^212^Pb by i.p. (Panels **A**–**D**) or i.v. (Panels **E**–**H**) injection. The effect on serum levels of ALT, AST, BUN and creatinine are shown. The error bars represent the standard deviations.

At necropsy, blood was collected for the chemistry analyses. Again, for the same reasons described earlier, data is only available for the 7 days (i.p. injection) at the 0.0925 to 1.110 MBq levels of activity. In general, clinical chemistry parameters remained within normal range, for the molecules of particular interest for this study, alanine aminotransferase (ALT), aspartate aminotransferase (AST), blood urea nitrogen (BUN) and creatinine. ALT and AST are indicators of hepatic disease while BUN and creatinine are used for monitoring renal health status. Elevations in any of these markers would indicate a reduction in hepatic or renal function. In the group of mice that received ^212^Pb by i.p. administration, the hepatic enzymes, AST and ALT, fluctuated and the changes do not appear to be activity-dependent (Figure 3A,B). At the 7 day timepoint, no significant differences were observed at any of the activity levels compared to the control group (*p* ≥ 0.231). At 90 days, the ALT was higher for those mice given 0.185, 0.278, 0.555, 0.740 and 1.110 MBq of ^212^Pb by the i.p route. However, the differences were not significant compared to the control group (*p* ≥ 0.076). Meanwhile, just as with the ALT, the level of AST at 7 days for the groups given i.p. ^212^Pb was lower than the control group. In contrast, at 90 days the AST was higher for all activity levels except the 0.555 MBq groups. Both of the hepatic enzymes, ALT and AST, were at their highest level in the 1.85 MBq i.p. injected group however the differences were not significant compared to the untreated group of mice (*p* = 0.113 for ALT and AST). In contrast, ALT levels in the mice given ^212^Pb by i.v. injection were the same at the 7 and 90 day timepoints at all levels of ^212^Pb activity. AST levels were elevated in the 7 day group that received 1.110 MBq of ^212^Pb; however, the difference was not statistically significant compared to the control group (*p* = 0.411). Interestingly, only one group (1.110 MBq) from the i.v. injected mice experienced a significant change in AST levels, compared to the control group, which occurred at the 90 day timepoint (*p* = 0.035). The AST levels in the rest of the i.v. injected mice were comparable to the untreated mice at both the 7 and 90 days timepoints (*p* ≥ 0.121).

The renal indicator, BUN, peaked in elevation with 0.185 and 0.278 MBq for the 7 and 90 day time points, respectively, in the mice given ^212^Pb by i.p. injection (Figure 3C). In fact, the 7 days BUN levels were above normal levels (compared to the untreated group) for the 0.0925 to 0.740 MBq i.p. injected ^212^Pb and below normal level for the 1.110 MBq group. This elevation was significant for mice administered the 0.185 to 0.555 MBq of ^212^Pb (*p* ≤ 0.009). At the 90 day time point, only the 0.185 and 0.278 MBq level of activity resulted in above normal BUN levels while the 0.740–1.850 MBq groups presented with lower levels of BUN which were still within the range of the control mice (*p* ≥ 0.079). Of these mice, euthanized at 90 days, only the group given 0.555 MBq had a significant difference in BUN levels compared to the untreated mice (*p* = 0.039). At 7 days (Figure 3D), creatinine levels for the mice receiving an i.p. injection of ^212^Pb, at all activity levels presented with values above those of the control group. The change in creatinine was significant for the groups receiving 0.185, 0.278, 0.370 and 0.555 MBq of ^212^Pb (*p* ≤ 0.022). At the 90 days timepoint, the differences were still notable for the mice injected i.p. with 0.278 MBq (*p* = 0.034) and 0.555 MBq (*p* = 0.017) of ^212^Pb as well as the group administered 0.740 MBq (*p* = 0.01).

**Figure 4:**
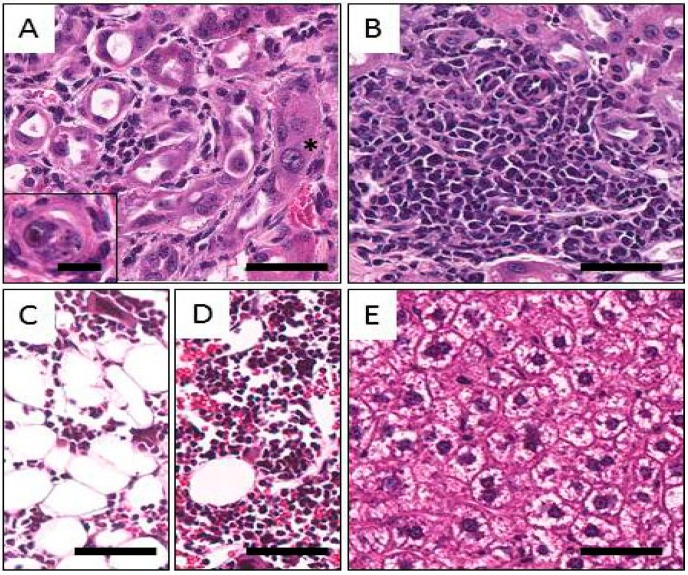
Representative images of histological findings following injection of Balb/c mice with ^212^Pb. **A**: Kidney; distorted tubular structures with macronuclei (star), mild fibrosis and mononuclear infiltrates. The inset shows a tubule with necrotic and possibly calcified cellular cast in the lumen. **B**: Kidney. Diffuse mononuclear infiltrate within an area of fibrosis. **C**: Bone Marrow. Hypoplastic bone marrow is shown in the right picture; notice the paucity of cellular elements and the relative increase in adipose tissue. **D**: Normal bone marrow for comparison. **E**: Liver. Cytoplasmic clearing can be seen the hepatocytes, possibly due to the accumulation of glycogen as a sign of mild chronic toxicity. The magnification bar in the inset to Figure 4A represents 20 µm, and all other bars 100 µm.

The pattern of the BUN and creatinine response for the i.v. injected mice differed (Figure 3G and Figure 4H) from that of the i.p. injected mice. The BUN values were lower than the control group at the 7 day time point for all activity levels with the exception of the 1.110 MBq. At the 90 day time point only the 0.0925 MBq group showed a significant difference in BUN levels (higher) compared to the control group (p = 0.047). Creatinine, at 7 days, was found to be significantly higher than the control mice for the groups injected with 0.185, 0.278 and 0.555 MBq of ^212^Pb. At the 90 day time point, the creatinine level was greater than the control group for those mice given 0.0925, 0.278, 0.370, 0.555 and 0.740 MBq.

### 2.5. Histological Findings Following Injection of Normal Balb/c Mice with ^212^Pb

Summarized in Table 3, treatment-related findings were found in the hematopoietic system, kidneys and liver. The ovaries, uterus, intestine, bladder, and lungs were examined with special care, but neither radiation nor lead toxicity was noted in these organs.

Activity-related findings were found in the bone marrow in all groups. Mild to moderate hypoplastic changes, with decreases in all series, erythroid, myeloid, and megakaryocytic, were prominent at 7 days following i.v. injection of 0.278 and 0.555 MBq, or i.p. injection of 0.278 and 0.740 MBq (Figure 4C). Mild splenic hematopoiesis was observed at 7 days in animals given 0.278 or 0.555 MBq; at 7 days at 0.185 MBq, i.p. and at 90 days at 0.740 MBq, i.p. Among the 90 day animals, bone marrow hypoplasia was noted at 0.555 MBq, i.p., but not at 0.740 MBq, i.v.

For the kidneys, at both 7 and 90 days, all groups (except 7 days at 0.185 MBq, i.p.) exhibited mild to moderate focal changes characterized by multifocal dilation of the cortical tubules; epithelial loss; hyperplastic and karyomegalic proliferation of epithelial cells; mild interstitial fibrosis; and mononuclear cell infiltration. Urinary casts were also observed. The observed changes are presumably associated with radiation. Although there appeared to be an activity-related trend for increases in incidence and severity, the lesions were not considered severe enough to have significantly affected kidney function. Representative images of these alterations are shown in Figure 4A,B.

**Table 3:** Correlation of treatment-related histopathologic findings with hematology following injection of Balb/c mice with ^212^Pb.

Injection Route	Activity (MBq)	Timepoint (day)	Kidneys (Chronic Nephritis)	Decreases in WBC and Platelet Counts	Bone Marrow Hypoplasia ^a^	Spleen (EMH, Hyperplasia of Red Pulp) ^b^	Increases in Liver Parameters	Hepatic Vacuolization
None	0	7	0/5	0	0/5	0/5	0	0
I.V.	0.185	7	3/5	0	0/5	0/5	0	1/5
	0.278	7	4/5	Yes	3/5	4/5	0	5/5
	0.555	7	1/5	Yes+	4/5	0/5	0	2/5
	0.555	90	5/5	0	0/5	4/5	0	2/5
I.P.	0.185	7	0/5	0	0/5	3/5	0	0
	0.278	7	1/5	Yes	5/5	4/5	0	0
	0.740	7	3/5	Yes	5/5	0/5	0	2/5
	0.740	90	5/5	Yes	5/5	2/5	0	4/5
	1.850^c^	90	2/2	ND ^d^	0/2	0/2	++	2/2

^a^ Includes bone marrow hypoplasia of any or all of the erythroid, myeloid and megakaryotic series. ^b^ Includes extramedullary hematopoiesis and/or hyperplasia of the red pulp, which are related to increased hematopoietic demand. ^c^ Only two animals from this group were submitted for histological examination. ^d^ ND, No data is available.

Mild multifocal to diffuse cytoplasmic clearing of hepatocytes was present in the livers of mice at 7 days after i.v. or i.p. injection at any of multiple levels of activity, as well as at 90 days after i.p. administration of 0.555, 0.740, or 1.850 MBq (Figure 4D). These changes were accompanied by corresponding increases in AST and ALT levels at 1.850 MBq at 90 days, indicating that a probable low-grade hepatic toxicity was present in these groups.

## 3. Discussion

As part of the process to obtain the authorization for the first ^212^Pb-labeled monoclonal antibody Phase 1 clinical trial, the FDA requested a toxicology study be performed with “free” ^212^Pb. The objective was to assess the short- and long-term effects of the radionuclide if there were a complete failure of the ^212^Pb to remain complexed with the immunoconjugate. The concern is understandable in that Pb(II) will localize in the kidneys, liver, erythrocytes and spleen [15,16,17,18]. In sum, the ^212^Pb injectate did have an impact on hepatic, renal and hemapoietic tissue. However, the likelihood of such an event in the clinic is remote for a number of reasons.

First and foremost, prior to the injection of patients, radioimmuno-conjugates (RIC) are subjected to several *in vitro* tests to insure retention of their integrity and bioactivity. During radiolabeling a chelate is added to the reaction mixture to scavenge remaining “free” radiometal, which is then removed in a purification step. The purified RIC is assayed in quality control procedures to determine if any free radiometal is in the injectate. Lastly, the chelate is added to the final formulation for the patient. Radiometals such as ^212^Pb will complex with the chelate and be eliminated from the body via renal excretion.

As mentioned earlier, the TCMC chelate was developed specifically for lead radioisotopes. The ^203^Pb[TCMC]^2+^ complex was found to be less labile to metal ion release than ^203^Pb[DOTA]^2−^ at pH ≤ 3.5 [14]. Greater stability for a ^203^Pb-labeled mAb using TCMC was also demonstrated. When incubated in serum, 83% of the radioactivity, remained associated with the mAb at 72 h which represents 6.8 half-lives of ^212^Pb [19].

Next to consider is the catabolism of the RIC, whether it is internalized by a tumor cell upon binding to its target or processed in non-target organs, *i.e.*, liver, spleen, and kidneys, for elimination. Studies have demonstrated that following internalization, radiometals accumulate in lysosomes [20,21]. Furthermore, when excretion of the radiometal does occur following lysosomal degradation it is as a low molecular weight metabolite, not generally as a free metal ion. Studies by Rogers *et al.* [22] demonstrated that the main excretory product following injection of an ^111^In-DTPA-monoclonal antibody was ^111^In-DTPA-ε-lysine. Other metabolic studies have reported similar results; the radiometal is retained in the chelate and excreted as the amino acid conjugate [23]. The data presented in this manuscript assuaged the FDA’s concern on the effects free ^212^Pb would have *in vivo*. In 2011, the IND was approved in January and the clinical trial was opened at UAB for accrual in July. The activity levels chosen for this study were selected to bracket and exceed the expected therapeutic dose in mice based on studies published from this laboratory [1,4,5,6]. The activity ranged from 0.925 to 1.850 MBq and 0.0925 to 1.110 MBq ^212^Pb for the i.p. or i.v. administration, respectively. All of the mice in the acute toxicity (7 days) groups survived to the scheduled time of euthanasia. Meanwhile, in the groups simulating chronic toxicity (90 days), deaths occurred in an activity-dependent manner in the sets of mice that received i.p. injections of 0.555, 1.110, 1.488 and 1.850 MBq of ^212^Pb and of the i.v. injected mice, deaths were recorded for only the 0.740 MBq group. These results are comparable to the maximum tolerated activity (MTA) determined for two intact mAbs labeled with ^212^Pb, trastuzumab (MTA between 0.740 and 1.488 MBq) and AE1 (MTA greater than 0.925 MBq), in two different models for HER-2 positive disseminated intraperitoneal disease using athymic mice [1,5]. In the latter study, non-tumor bearing mice were injected i.v. with 0.370, 0.925 or 1.488 MBq of ^212^Pb-AE1 and monitored for toxicity. Hematological and serum chemistry parameters were measured over a 180 days period. Mice injected with 1.488 MBq of ^212^Pb-AE1 experienced severe weight loss (25.8%) and significant lymphoid depletion and died within 6–10 days. The greatest weight loss noted in the current study was 10.3% at 7 days in the group administered 0.740 MBq by i.p. injection. Mice receiving the 0.370 and 0.925 MBq of ^212^Pb-AE1 experienced transient decreases in WBC, platelets and lymphocytes with a nadir ranging from 4–11 days with recovery of cell counts by 60 days. Again, ^212^Pb alone had a similar effect on blood cell counts at the lower activity levels; however, at higher activity (>0.555 MBq), cell counts did not return to normal levels by 90 days in most of the groups. There was a large variance in values within groups suggestive of differences in individual animal recovery, ranging from a poor recovery to a hyperplastic rebound effect. There was not an apparent correlation between activity level and hematologic recovery. For example, in the mice given the ^212^Pb by i.p. injection, the PMNs of the 0.370 MBq group do not recover by 90 days while the in the 1.110 and 1.488 MBq there is a rebound. Again this may be due to differences in the recovery of individual animals. The noted nadir of blood counts which occurs at ~10 days would be more accurate (sooner for high levels of radioactivity) if the blood collection had been performed more frequently in the first two weeks.

In the ^212^Pb-AE1 study, BUN and creatinine were found to be elevated in 75% of the mice injected with 0.925 MBq of the RIC suggesting the presence of a chronic nephritis. Increases of these two indicators of renal disease were observed following the i.p. injection of ^212^Pb alone after 7 days. The BUN levels returned to normal levels by 90 days while the creatinine remained elevated at 0.555 MBq and higher in those mice given the ^212^Pb via i.p. injection. Among the groups receiving the ^212^Pb by the i.v. route, BUN levels were elevated in the 1.110 MBq group at 7 days while all of the groups demonstrated elevated creatinine levels at 90 days with the exception of the groups given 0.185 and 1.110 MBq. Herein, chronic nephritis was confirmed by histopathology. Among the mice administered the ^212^Pb by i.p. injection, only the 0.185 MBq group appeared free of nephritis. A greater number of mice exhibited nephritis at 7 days in the i.v. injected groups selected for analysis. In contrast to the study with ^212^Pb-AE1, several of the mice in both the i.p. and i.v. groups exhibited hyperplasia of the spleen red pulp.

In this study, of the groups examined, mild to moderate bone marrow hypoplasia was noted at 7 days in all of the groups of mice. Interestingly, at 90 days the bone marrow hypoplasia was present in the 0.555 MBq (i.p.) group and yet not the 0.740 MBq or 1.850 MBq (i.v) groups. This result suggests that that the bone marrow received a greater dose with the i.p. injected ^212^Pb.

The ^212^Pb, with either i.p. or i.v. injection routes, also had an impact on the livers of the mice as would be expected by clearing of ^212^Pb by hepatocytes. The effect was noted to various degrees in all of the i.v. groups examined histologically and in the groups given 0.740 and 1.850 MBq i.p. The elevated ALT and AST correlated with the visual hepatic findings suggesting a low-grade toxicity. Agents injected into the peritoneum such as radiolabeled antibodies do redistribute out of the peritoneal cavity into the blood.[24,25] The effect of ^212^Pb given i.p. on hepatocytes would most likely not be as dramatic as what was observed in the i.v. injected mice for two reasons. First, not all of the i.p. injected ^212^Pb would enter the blood compartment. More importantly, however, the redistribution from the peritoneum to the blood takes time, and with time, the radioactivity entering and circulating in the blood is reduced as is the toxicity to organs such as the liver.

The clinical trial at UAB, expanded to the Moores Cancer Center at the University of California, San Diego where the final cohort of patients were treated in October of 2014. Preliminary reports have shown promising results [24,26]. Minimal toxicity was reported for the first five dosage levels (7.4–21.1 MBq/m^2^; 0.2–0.57 mCi/m^2^), given i.p., for at least 4 months and up to 1 year for the first cohort of patients [26]. Limited redistribution of the radioactivity out of the peritoneal cavity into the circulating blood occurred, which was then cleared via urinary excretion. At 18–24 h post-injection of the ^212^Pb-TCMC-trastuzumab, no specific uptake of radioactivity was observed in the major organs by whole body γ-camera imaging [24].

Interest in α-particle radiation therapeutics has long been pursued and now several clinical trials have been performed with either ^213^Bi, ^211^At, and now ^212^Pb radiolabeled mAbs targeting various cancers. Use of ^225^Ac radiolabeled mAb targeting CD33s has proceeded to multi-center status. However, perhaps more important is the 2013 precedent-setting FDA approval of ^223^Ra dichloride (Xofigo), an α-emitting radionuclide, for the treatment of patients with castration-resistant prostate cancer, symptomatic bone metastases and no known visceral metastatic disease. The radium mimics calcium and forms complexes with hydroxyapatite at areas of increased bone turnover, such as bone metastases. As such, this action has spurred considerable interest in other targeted α-therapy agents that have been reported in the literature and may promote further translation of these agents into clinical evaluation.

## 4. Experimental Section

### 4.1. Preparation of ^212^Pb and Sampling

The ^212^Pb, eluted from ^224^Ra generators supplied by Areva Med LLC (Bethesda, MD, USA) has been previously described. Briefly, the ^224^Ra generators (74–185 MBq) were washed with 2 M HCl to remove impurities, unbound ^224^Ra, daughter isotopes, and any damaged resin or organic residue. On the following day, ^212^Pb was eluted from the generator with 2 M HCl. The eluate was then heated to dryness and the residue digested three times with nitric acid (8 M) and the dry residue dissolved in 0.1 M HNO_3_. The nitric acid solution was first neutralized to pH 6 with an ammonium acetate solution (5 M) and diluted appropriately in normal saline for injection of the mice. A calibrated Ge(Li) detector (model GEM10185-P; EG&G Ortec, Oak Ridge, TN, USA) coupled to a multichannel analyzer and a computer running Gamma Vision version 5.2 software (EG&G Ortec) was used for radioactivity measurements. The ^212^Pb activity was determined by measurement of the 238.6 keV γ-ray. Activities were based on the time of injection.

### 4.2. Animal Model

Studies were conducted with 5–6 week old Balb/c mice (Frederick National Laboratory for Cancer Research, Frederick, MD, USA). As outlined in Table S4, groups of mice received ^212^Pb by either intraperitoneal (i.p., 0.0925–1.850 MBq) or intravenous (i.v., 0.0925–1.110 MBq) injection. One group of mice was left untreated. Five mice from each treatment group were used exclusively for hematology analysis. The remainder of the treatment group was entered into either a 7 or 90 days observation period. At the end of the observation period, mice were taken to the laboratory and euthanized by CO_2_ narcosis for blood collection and necropsy. Based on previous reports from this laboratory, the range of radioactivity was selected to encompass and exceed the expected therapeutic dose as calculated based on murine body area. All animal studies were conducted under an animal protocol approved by the National Cancer Institute Animal Care and Use Committee.

### 4.3. Measurements

#### 4.3.1. Body Weight

The weights of the mice were recorded before injection of the ^212^Pb and at the end of the observation period (7 or 90 days) immediately before euthanasia.

#### 4.3.2. Hematology

In each treatment group, one set of 5 mice per group was used exclusively for hematology determinations. Blood (150 μL) was collected via an intravenous or intra-arterial catheter placed in the tail on days 7, 14, 28, 56 and 90. Five untreated mice were used as controls. At collection, the blood was diluted with 150 μL 1.5 mg/mL potassium ethylenediamine tetraacetic acid (K-EDTA) in phosphate buffered saline (PBS). Blood samples were submitted for analysis within 8–10 h of collection to the Department of Laboratory Medicine, NIH Clinical Center, which is in compliance with the College of American Pathologist regulations and guidelines. The hematology analysis included red blood cell (erythrocyte) count, white blood cell (leukocyte) count, platelet count, hemoglobin, hematocrit, mean corpuscular volume and differential blood cell count (absolute and %).

#### 4.3.3. Clinical Chemistry

Groups of 5 mice were used for both blood chemistry analyses and for necropsy. For the control group, a group of 5 untreated normal control mice was used. In each treatment group, blood samples (0.5–1 mL) for clinical chemistries were collected with no anticoagulant following aortic transection. Samples were allowed to clot at room temperature for 15 min, and the serum was separated and transferred to clear polypropylene tubes. The serum samples were submitted for analysis within 4–6 h of collection to the Department of Laboratory Medicine, NIH Clinical Center. Eleven clinical chemistry parameters were measured with a focus on markers for hepatic and renal health status. Alanine aminotransferase (ALT), alkaline phosphatase, and aspartate aminotransferase (AST) are indicators of hepatic dysfunction. Blood urea nitrogen (BUN), calcium, and creatinine are markers of renal disorder. Albumin and total protein prvode information for both liver and kidney functioning. Glucose, inorganic phosphorus and total cholesterol provide general health and metabolic information.

#### 4.3.4. Histopathology

Complete necropsies were performed and samples from all organs extracted (Table S5) following standard guidelines (Revised guides for organ sampling and trimming in rats and mice, Parts 1, 2, and 3 [27]. The tissues were gross trimmed, fixed in neutral formalin for 24 h, and embedded in paraffin. Forty-seven mice from the 10 treatment groups, indicated in Table S6, were selected for histopathological examination based on the hematology data by a board-certified veterinary pathologist. The preserved tissues (Table S6) were submitted to the CBI Histology Laboratory (Fort Worth, TX, USA). Five-micron sections were prepared, stained with hematoxylin-eosin and examined microscopically.

### 4.4. Statistical Analysis

Results are expressed as the average mean ± the standard deviation. Statistical differences between the groups were determined using a 2-tailed Student t-test test. Statistically significant difference between datasets was determined at *p* < 0.05. Statistical analysis was performed using SigmaPlot version 12.5 software (SysStat Software, Inc., San Jose, CA, USA).

## Supplementary Files

Supplementary File 1

## Abbreviations

mAb, monoclonal antibody; RIT, radioimmunotherapy; TCMC, 1,4,7,10-tetra-(2-carbamoyl methyl)-cyclododecane;FDA, Food and Drug Administration; i.p., intraperitoneal; i.v., intravenous; ND, no data; WBC, white blood cells; PMN, polymorphonuclear; RBC, red blood cell; ALT, alanine aminotransferase; AST, aspartate aminotransferase; BUN, blood urea nitrogen; RIC, radioimmunoconjugate; MTA, maximum tolerated activity.
