# Supplementary material for: Toxicological Studies of 212Pb Intravenously or Intraperitoneally Injected into Mice for a Phase 1 Trial
Source: Pharmaceuticals (Basel). 2015 Jul 24;8(3):416–34. doi: 10.3390/ph8030416 (PMC4588175; doi:10.3390/ph8030416)
Supplement: Supplementary File 1 [file pharmaceuticals-08-00416-s001.pdf]

Supplementary Materials

Table S1. Time course of blood cell counts following the i.p. injection of Balb/c mice (n = 5) with 0.0925 to 1.850 MBq of ^{212}Pb, bloods were drawn weekly and analyzed.

Cell	MBq	Weeks Post Injection of ^{212}Pb													
		0		1		2		3		4		8		12	
		Aver	St Dev	Aver	St Dev	Aver	St Dev	Aver	St Dev	Aver	St Dev	Aver	St Dev	Aver	St Dev
WBC K/μL	0.093	7.06	2.22	4.9	1.8	8.6	2.29			9.47	3.55	7.39	2.78	9.29	4.44
	0.185			4.78	2.36	4.26	0.83			5.44	1.94	4.73	1.36	6.49	2.16
	0.278			2.76	0.62	1.72	1.75			4.32	0.92	4.72	1.49	4.56	2.4
	0.370			2.38	0.69			6.1	1.86	3.93	1.26			8.78	3.88
	0.555			1.46	0.59			3.43	0.41	3.22	1.32			3.94	1.26
	0.740			0.61	0.28			4.79	2.33	3.84	2.45			4.89	1.02
	1.110			0.42	0.26	2.21	1.4			2.93	1.82	5.11	2.88	4.77	3.02
	1.488			0.31	0.22	2.14	1.71			3.37	1.03	5.86	1.55	6.39	0.98
	1.850			0.4	0.37	1.53									
Platelets K/μL	0.093	1060	230	892	122	1207	190			1158	107	1012	248	1060	261
	0.185			769	109	1056	21			1108	123	917	236	1238	92
	0.278			505	60	978	285			1031	111	1010	206	1075	223
	0.370			284	59			745	211	764	238			1018	450
	0.555			238	65			873	163	803	140			787	248
	0.740			99	49			671	198	582	99			792	222
	1.110			118	31	586	186			655	368	753	454	778	448
	1.488			136	69	375	462			755	183	949	144	1035	147
	1.850			127	16	402									

Table S1. *Cont.*

**Cell**	**MBq**	**Weeks Post Injection of ^{212}Pb**													
		**0**		**1**		**2**		**3**		**4**		**8**		**12**	
		**Aver**	**St Dev**	**Aver**	**St Dev**	**Aver**	**St Dev**	**Aver**	**St Dev**	**Aver**	**St Dev**	**Aver**	**St Dev**	**Aver**	**St Dev**
	0.093	1.48	0.75	1.15	0.93	0.57	0.4			2.2	1.01	1.24	0.81	1.29	1.24
	0.185			0.703	0.71	0.39	0.65			0.74	0.8	0.96	0.63	0.91	1.14
	0.278			0.4	0.42	0.75	0.4			1.4	0.83	1.3	0.57	0.58	1.15
PMN K/μL	0.370			0.16	0.21			1.69	0.43	1.06	0.45			1.03	1.11
	0.555			0.06	0.13			1.02	0.52	1.59	1.24			0.76	0.51
	0.740			0.02	0.01			2.14	0.3	1.32	0.65			0.49	0.55
	1.110			0.01	0	0.35	0.55			0.65	0.82	1.22	1.01	2.1	1.63
	1.488			0.01	0	0.87	0.68			1.79	1.51	1.18	1.4	1.39	0.86
	1.850			0.02	0.02										
	0.093	10.79	0.93	10.21	0.78	10.56	0.7			11.13	0.65	9.56	0.83	9.14	0.62
	0.185			10.61	1.45	9.82	0.74			10.24	1.06	9.99	0.79	9.71	0.75
	0.278			9.68	0.62	9.96	0.45			10.54	1.08	11.24	1.3	8.64	1.66
RBC M/μL	0.370			10.08	0.5			10.58	1.66	11.94	1.48			10.88	3.82
	0.555			9.5	0.58			10.02	0.37	10.19	0.99			10.83	1.73
	0.740			9.1	0.86			8.46	2.98	10.23	0.4			9.01	0.82
	1.110			8.74	0.83	7.72	0.99			8.02	4.51	8.02	4.44	7.92	4.54
	1.488			10.02	1.32	8.04	2.17			9.82	0.75	10.02	0.98	9.04	1.12
	1.850			13.59	5.39	8.08									

Table S2. Time course of blood cell counts following the i.v. injection of Balb/c mice (*n* = 5) with 0.0925 to 1.850 MBq of ^{212}Pb, bloods were drawn weekly and analyzed.

**Cell**	**MBq**	**Weeks Post Injection of ^{212}Pb**													
		**0**		**1**		**2**		**3**		**4**		**8**		**12**	
		**Aver**	**St Dev**	**Aver**	**St Dev**	**Aver**	**St Dev**	**Aver**	**St Dev**	**Aver**	**St Dev**	**Aver**	**St Dev**	**Aver**	**St Dev**
WBC K/µL	0.093	7.06	2.22	3.588	1.348	4.75	1.83			4.53	1.25	4.51	1.33	4.894	1.864
	0.185			5.82	1.17	4.52	1.13			4.91	1.28	4.46	1.11	5.68	2.728
	0.278			2.83	1.291	2.97	2.25			5.73	1.49	6.59	1.62	5.425	2.283
	0.37			0.9	0.096			4.6	2.52	4.49	0.83			4.982	1.6
	0.555			0.91	0.131			3.85	1.02	4.78	1.28			8.08	1.41
	0.74			0.326	0.195			0.33	0.2	4.75	1.39			1.905	0.8
	1.11			0.334	0.356	0.51	0.45			6.74	3.37	5.8	1.45	6.98	2.19
Platelets K/µL	0.093	1060	230	798	78	971	175			918	170	828	53	991	183
	0.185			784	99	1302	88			973	271	984	152	1163	319
	0.278			557	109	775	241			909	338	982	152	1135	335
	0.37			152	28			631	177	619	103			865	468
	0.555			111	26			671	242	438	293			755	176
	0.74			105	52			105	52	675	99			729	196
	1.11			130	36	79	43			703	113	781	127	1020	100
PMN K/µL	0.093	1.48	0.75	0.68	0.16	0.58	0.58			1.14	0.43	0.91	0.61	0.473	0.683
	0.185			1.36	0.68	0.25	0.1			1.42	0.61	0.79	0.58	1.118	1.07
	0.278			0.603	0.06	0.37	0.41			1.57	0.83	1.6	0.93	1.331	1.243
	0.37			0.023	0.034			3.37	2.44	2.01	1.1			1.276	0.798
	0.555			0.014	0.005			2.07	0.51	0.85	0.72			1.1	0.79
	0.74			0.012	0.009			0.01	0.01	1.29	0.32			0.2	0.04
	1.11			0.006	0.002	0.01	0.01			3.8	3.8	1.26	0.53	1.02	1.549

Table S2. *Cont.*

Cell	MBq	Weeks Post Injection of ^{212}Pb													
		0		1		2		3		4		8		12	
		Aver	St Dev	Aver	St Dev	Aver	St Dev	Aver	St Dev	Aver	St Dev	Aver	St Dev	Aver	St Dev
RBC M/µL	0.093	10.79	0.93	10.1	0.67	9.38	0.97			10.68	1.07	10.34	0.94	10.06	0.62
	0.185			11.1	1.38	10.08	0.52			10.53	2.06	10.48	0.47	10.05	0.85
	0.278			9.99	0.49	10.46	1.2			10.48	2.12	10.96	0.38	9.66	1.06
	0.37			9.29	0.36			9.88	0.76	10.94	0.51			12.46	5.43
	0.555			9.27	0.51			10.27	0.46	7.92	4.41			11.57	1.08
	0.74			9.28	0.26			9.28	0.26	10.3	0.71			10.93	1.82
	1.11			8.89	0.89	6.36	1.43			9.55	0.6	10.09	0.85	8.34	0.45

Table S3. Hematological analysis of untreated mice.

		Weeks							
		0		4		8		12	
		Average	St Dev	Average	St Dev	Average	St Dev	Average	St Dev
WBC	K/uL	5.90	1.98	9.20	2.57	5.86	0.88	7.27	1.66
RBC	M/uL	9.94	0.52	11.44	0.59	10.21	0.57	11.55	0.74
Platelets	K/uL	970.00	126.59	1219.40	91.31	946.00	277.77	1104.40	298.82
PMNS	K/uL	1.74	0.21	1.15	0.85	1.89	0.78	1.15	0.88

Table S4. Summary of experimental design.

Injection Route	No. of Mice	Activity Level (MBq)
None	15	0
Intraperitoneal	15	0.0925
	15	0.185
	15	0.278
	20	0.370
	20	0.555
	15	0.740
	15	1.110
	9	1.480
	10	1.850
Intravenous	15	0.0925
	20	0.185
	15	0.278
	20	0.370
	15	0.555
	15	0.740
	15	1.110

Table S5. Tissues harvested at necropsy.

Adrenal Glands (2)	Heart	Rectum
Abdominal adipose	Ileum	Salivary glands
Brain (brain stem, cerebrum, cerebellum)	Jejunum	Skin
Cecum	Kidneys (2)	Spleen
Colon	Liver	Stomach
Duodenum	Lung	Thymus
Esophagus	Lymph node (mesenteric)	Thyroid
Eyes	Muscle (quadriceps)	Trachea
Femur with bone marrow	Ovaries (2)	Urinary Bladder
Gall Bladder	Pancreas	Uterus

Table S6. Summary of histopathology experimental design.

Administration Route	No. of Mice	Activity (MBq)	Histopathologic Examination	
			7 Days	90 Days
None	15	0	X	
i.p.	15	0.0925		
	15	0.185	X	
	15	0.278	X	
	20	0.370		
	20	0.555	X	X
	15	0.740		
	14	1.110		
	10	1.480		
	10	1.850		X
i.v.	15	0.0925		
	20	0.185	X	
	15	0.278	X	
	20	0.370		
	15	0.555	X	X
	15	0.740		
	15	1.110		

n = 5 for each group examined. The control group was a group of untreated normal mice that did not receive vehicle.

© 2015 by the authors; licensee MDPI, Basel, Switzerland. This article is an open access article distributed under the terms and conditions of the Creative Commons Attribution license (http://creativecommons.org/licenses/by/4.0/).
